# Detection of Genes Related to Antibiotic Resistance in *Leptospira*

**DOI:** 10.3390/tropicalmed9090203

**Published:** 2024-09-06

**Authors:** Santiago Pineda, Juliana María Martínez Garro, Jorge Emilio Salazar Flórez, Sergio Agudelo-Pérez, Fernando P. Monroy, Ronald Guillermo Peláez Sánchez

**Affiliations:** 1 CES Biology, Science and Biotechnology School, CES University, Medellin 050021, Colombia; pineda.gomez@uces.edu.co (S.P.); jmartinezg@ces.edu.co (J.M.M.G.); 2 Medicine Program, GEINCRO Research Group, School of Health Sciences, San Martín University Foundation, Sabaneta 055450, Colombia; jorge.salazarf@sanmartin.edu.co; 3 Department of Pediatrics, Medicine School, Universidad de La Sabana, Chía 025001, Colombia; sergioagpe@unisabana.edu.co; 4 Department of Biological Sciences, Northern Arizona University, Flagstaff, AZ 86011, USA; fernando.monroy@nau.edu; 5 Life and Health Sciences Research Group, Graduate School, CES University, Medellin 050021, Colombia

**Keywords:** genomes, sequences, microbicidal, pathogens, saprophytes

## Abstract

Leptospirosis is a disease caused by the bacteria of the *Leptospira* genus, which can usually be acquired by humans through contact with urine from infected animals; it is also possible for this urine to contaminate soils and bodies of water. The disease can have deadly consequences in some extreme cases. Fortunately, until now, patients with leptospirosis have responded adequately to treatment with doxycycline and azithromycin, and no cases of antibiotic resistance have been reported. However, with the extensive use of such medications, more bacteria, such as *Staphylococci* and *Enterococci,* are becoming resistant. The purpose of this study is to determine the presence of genes related to antibiotic resistance in the *Leptospira* genus using bioinformatic tools, which have not been undertaken in the past. Whole genomes from the 69 described *Leptospira* species were downloaded from NCBI’s GeneBank and analyzed using CARD (The Comprehensive Antibiotic Resistant Database) and RAST (Rapid Annotations using Subsystem Technology). After a detailed genomic search, 12 genes associated with four mechanisms were found: resistance to beta-lactamases, vancomycin, aminoglycoside adenylyltransferases, as well as multiple drug efflux pumps. Some of these genes are highly polymorphic among different species, and some of them are present in multiple copies in the same species. In conclusion, this study provides evidence of the presence of genes related to antibiotic resistance in the genomes of some species of the genus *Leptospira*, and it is the starting point for future experimental evaluation to determine whether these genes are transcriptionally active in some species and serovars.

## 1. Introduction

The *Leptospira* genus consists of bacteria that belong to the family *Leptospiracae* in the order of Leptospirales, class Spirochaetia, and phylum Spirochaetota [1,2,3,4,5]. At present, the *Leptospira* genus comprises 69 species, divided into two groups: the infectious group, divided into two subclades, P1, with 20 species, and P2, with 21 species; and the non-infectious group, which includes non-pathogenic or saprophytic organisms, divided into two subclades, S1 with 23 species, and the most recently proposed group, S2 with 5 species. This classification includes the four new species described in 2021 in Algeria: *L. ainlahdjerensis, L. ainazelensis, L. abararensis*, and *L. chreensis*, as well as the most novel species isolated from soil in Puerto Rico, *L. sanjuarensis*, described as a pathogen [6,7]. The leptospiral genome is distributed in circular chromosomes: a large one, chromosome I, and a small one, chromosome II, with an approximate length range between 3500–4300 KB and 300–350 KB, respectively. Saprophytic *Leptospira* also has a third circular replicon called p74, which has been linked to their survival as free-living organisms [8,9,10,11,12]. Pathogenic *Leptospira* causes a systemic disease called leptospirosis, a zoonic disease acquired by humans through direct or indirect contact with the urine of infected animals, wet soils, and environmental water sources contaminated with the bacteria [13]. In addition, different serovars are usually associated with specific hosts, making it important to identify possible animal sources of infection [2,14,15,16,17,18,19,20,21,22,23,24,25]. Human leptospirosis has emerged as a globally important infectious disease, happening mostly in urban and rural areas of developing countries, with a higher presence in the tropics. The expansion of the disease in these conditions is largely due to insufficient rapid diagnosis and poor sanitary measures [2,26,27,28]. A great portion of infections cause subclinical severity, in which case, patients do not seek medical attention. This type of leptospirosis is known as anicteric, and it might cause meningitis, although fewer than 25% of all cases of meningitis are caused by anicteric leptospirosis. Most cases of leptospirosis do not require hospitalization, and recovery tends to be spontaneous. However, the use of antibiotics is a common practice to prevent disease progression. In these cases, the use of second-generation antibiotics, such as doxycycline or azithromycin, is recommended. In more severe cases, broad-spectrum antibiotics such as penicillin, ampicillin, ceftriaxone, or cefotaxime are often used [2,28]. On the other hand, icteric leptospirosis is a more severe version of the disease that progresses very rapidly, and its death rate varies from 5 to 15%. Up to 15% of leptospirosis cases are considered icteric [29,30,31,32].

There have been efforts to study whether *Leptospira* would be susceptible to antibiotics against Gram-positive or Gram-negative bacteria, given the structure of its cell wall. In fact, the antibiotics that are usually used in anicteric cases work against Gram-positive bacteria, although the cell wall resembles that of Gram-negative bacteria. Therefore, over the years, different antibiotics have been tested on *Leptospira* to determine how this bacterium reacts to different treatments. A. Schönberg was the first person to analyze the effect of antibiotics (used for both Gram-positive and Gram-negative) on five *Leptospira* strains back in 1980, where vancomycin and nalidixic acid had the lowest effect while streptomycin and chloramphenicol showed the highest bacterial reduction [33]. In 1988, the University of Fukuyama analyzed the effects of streptomycin and other antibiotics on *L. biflexa*, one of the non-pathogenic species, and on a streptomycin (SM)-resistant mutant from the same species. The results showed that the SM-resistant mutant was also cross-resistant to dihydrostreptomycin [34]. A study in 2010 from Japan analyzed the in vitro susceptibly and resistance of 46 *Leptospira* strains against 14 different antibiotics, showing susceptibility to streptomycin and six other antimicrobial agents and resistance to vancomycin and six other antibiotics [35]. In 2018, five different antibiotics were tested on different strains of *L. santarosai*, *L. noguchii*, and *L. interrogans*, isolated from cattle in Brazil, showing susceptibility to tetracycline [36]. Another two studies in 2020 analyzed antibiotic susceptibility from 100 environmental, livestock, and human isolates, showing resistance to trimethoprim and sulphamethoxazole and susceptibility to penicillin, ceftriaxone, doxycycline, tetracycline, and six other antibiotics [37,38]. These studies from all over the world have tested different antimicrobial agents, including Gram-positive and Gram-negative, given the nature of *Leptospira*, which has characteristics of both stains. These studies have suggested that *Leptospira* might be resistant or at least susceptible to certain antibiotics, either via gene resistance or simply because they are not suited for the leptospiral structure. However, there have been limitations in the number of species and serovars used in the studies. A study that involves the isolation of all 69 species and every possible serovar is time-consuming, and the studies that have used the in vitro approach used only a few species and serovars. As a result, a more comprehensive study using other methods rather than direct exposure to the antibiotic is needed. Therefore, the purpose of this research is to investigate whether any *Leptospira* species across the genus has acquired such genes by analyzing the entire genome using bioinformatic tools.

## 2. Materials and Methods

### 2.1. Reference Genomes for the 69 Species of the Leptospira Genus

A representative genome of each of the 69 species of the genus *Leptospira* was downloaded from the NCBI-Genome database (https://www.ncbi.nlm.nih.gov/datasets/genome/). These genomes were grouped according to the species groups of the genus (P1, P2, S1, and S2). The sequences of the two chromosomes (three in some saprophytic species) of each species were concatenated with the (CAT) command of the Linux operating system terminal to obtain a continuous linear sequence (Ubuntu 24.04 LTS). The genomes were organized by species groups, and the genomic characteristics were discovered (species names, clade, accession number, genome size (Mb), GC content (%), genes, proteins, and non-coding sequence). The genomic characteristics of the different species belonging to the *Leptospira* genus are compared.

### 2.2. Detection of Genes Related to Antibiotic Resistance

#### 2.2.1. CARD (The Comprehensive Antibiotic Resistance Database)

The concatenated sequences of each of the 69 species of the *Leptospira* genus were analyzed using the web server CARD (The Comprehensive Antibiotic Resistance Database, version 3.2.4.) with the aim of identifying genes related to the antibiotic resistance mechanisms described in the *Leptospira* genus and other bacterial genera. Each sequence was analyzed independently, and the results of the 69 species were consolidated in a database using the Excel program (Microsoft^®^—Microsoft 365). The results were analyzed, and the genes were grouped according to the mechanism and type of drug to which they conferred resistance. This software is an attempt to consolidate all data describing the antibiotics and their targets, antibiotic resistance genes, and proteins. This database is linked to GeneBank and tagged with NCBI’s Taxonomy Ontology, which is associated with peer-reviewed scientific publications with clear experimental evidence of an elevated minimum inhibitory concentration (MIC) over the controls. RGI (Resistance Gene Identifier) is a tool in CARD that analyzes raw data using a BLAST, which is constantly curated [39,40,41,42,43,44].

#### 2.2.2. RAST (Rapid Annotations using Subsystem Technology)

The concatenated sequences of each of the 69 species of the *Leptospira* genus were analyzed using the web server RAST (Rapid Annotations using Subsystem Technology) (https://rast.nmpdr.org/rast.cgi) with the aim of identifying genes related to the antibiotic resistance mechanisms described in the *Leptospira* genus and other bacterial genera. Each sequence was analyzed independently, and the results of the 69 species were consolidated in a database using the Excel program. The results were analyzed, and the genes were grouped according to the mechanism and type of drug to which they conferred resistance. This is a service that aims to determine gene functions and metabolic pathways by comparing the existing annotated genomes. The results of the server are based on subsystems and protein families derived from the latter that are manually curated to produce assertions, with the latter being the basis for the metabolic reconstructions and gene functions maintained within the SEED integration [45,46,47].

### 2.3. Comparative Analysis of Genes Associated with Antibiotic Resistance among Leptospira Species

The genes associated with antibiotic resistance found using the CARD and RAST web servers were grouped for each *Leptospira* species. Subsequently, the identified genes were compared between the different species, and the results were presented in a heat map using the R (R version 4.4.1—Race for Your Life) and R study (version 2024.04.2+764) programs. Additionally, the packages (heatmap, heatmap.2, Pheatmap, d3heatmap, and CompletHeatmap) were used. This software provides a flexible solution for organizing and annotating multiple heat maps. It also allows for visualizing the association between different data from different sources. The number of copies found for each of the 12 genes associated with the four antibiotic resistance mechanisms was analyzed in each of the species.

### 2.4. Identification of the Biological Function of Genes

Using the BLAST algorithm of the NCBI database and the sequences of the genes associated with the antibiotic resistance mechanisms, the sequences of the encoded proteins were identified and downloaded. Using the protein sequence and the InterProScan bioinformatics program, the protein domains, biological processes, molecular function, and cellular components of the different proteins were identified. Finally, the biological information available for each protein in the Panther GO Terms and GeneCards databases was consulted. In this way, information on the different genes, proteins, and antibiotic resistance mechanisms was collected.

## 3. Results

### 3.1. Leptospira Species

Within the subclade, P1, *L. adleri*, *L. borgpetersenii*, *L. mayottensis*, and *L. santarosai* have the smallest genome, measuring 4 Mb, while *L. stimsonii* has the largest genome, measuring 5 Mb. Within subclade P2, *L. fletcheri* and *L. fluminis* have the smallest genome with 3.7 Mb, and *L. inadai* has the largest with 4.6 Mb. Meanwhile, the shortest genome in clade S1 belongs to *L. kemamanensis,* with 3.8 Mb, and the largest to *L. chreensis,* with 4.5 Mb. Finally, in subclade S2, the shortest genomes can be found in *L. ognonensis* and *L. ryugenii* with 4 Mb, while the largest is found in *L. kobayashii* with 4.3 Mb. On average, the P1 species genomes are the largest of all subclades, even above the average of all 69 species. The number of genes annotated also corresponds to the trend of the length of the genomes with subclade P1, with the most average genes at 4001, while P2, S1, and S2 have 3812, 3870, and 3844 genes, respectively. However, the number of protein-coding sequences is more conserved, with 3785, 3750, 3797, and 3775 in P1, P2, S1, and S2, respectively. Finally, the GC content is not as variable, with the smallest percentages present in *L. interrogans* in P1, *L. koniambonensis* in P2, *L. bouyouniensis* in S1, and *L. ognonensis* in S2, with 35, 38.5, 37, and 39.5, respectively. On the other hand, the largest percentages were present in *L. ellisii* in P1, *L. fluminis* in P2, *L. chreensis* in S1, and *L. idonii* in S2, with 47.5, 47.5, 39.5, and 41, respectively. The average GC content of all species was found to be 40.48 (Table 1).

### 3.2. Resistance to Vancomycin

**Figure 1:**
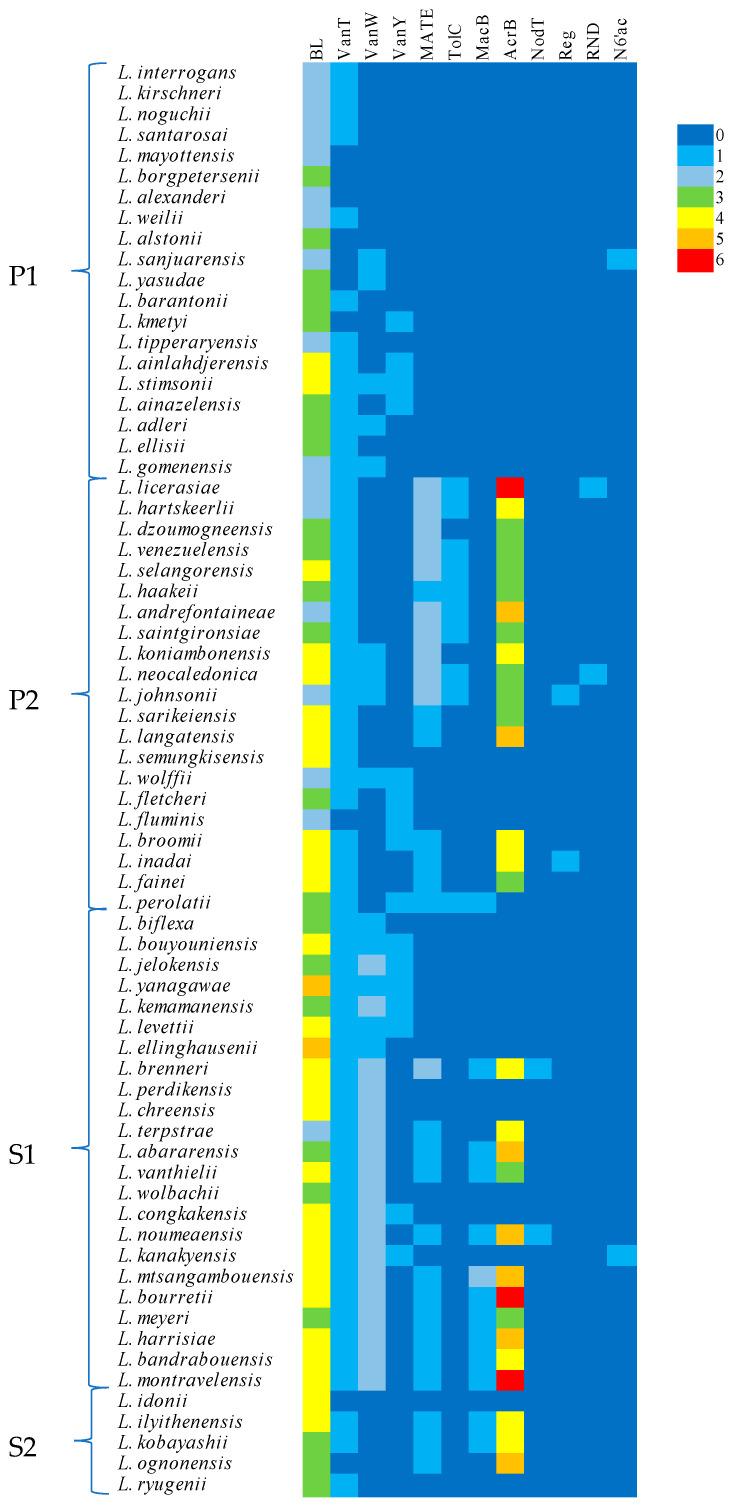
All 69 described *Leptospira* species discriminated by clade (P1, P2, S1, and S2) with a heat map where the number of copies found for each of the 12 genes associated with the four antibiotic resistances is shown: 1: beta-lactamase (BL); 2: vancomycin (VanT: vancomycin VanT gene in VanG cluster; VanW: vancomycin VanW gene in VanB and VanG clusters; VanY: vancomycin VanY gene in VanB and VanG clusters). 3: multidrug resistance efflux pumps (MATE: multidrug and toxic compound extrusion proteins; TolC: efflux protein; MacB: macrolide export ATP-binding protein; AcrB: acriflavine resistance protein; NodT: NodT protein; Reg: transcription regulators; RND: membrane fusion protein); and 4: aminoglycoside adenylyltransferases (N6′ac: aminoglycoside N6′-acetyltransferase).

The CARD analysis of the 69 genomes yielded five different genes associated with antibiotic resistance to vancomycin, present in 91% of species. The most common cassette present in *Leptospira* was the VanT gene in the VanG cluster, with a presence in 85.5% of all analyzed species. The second most common gene was VanW, present in two different clusters, VanB and VanG, with a 15.9% and 57.9% incidence, respectively. Finally, the least represented cassette is the VanY, with a 20.3% and 4.3% incidence in the VanB and VanG clusters, respectively. Although the VanW cassette appeared in two different clusters, they are homologs of the same gene, which can also be said for the VanY gene; therefore, they are represented as VanW and VanY (Figure 1).

The VanT gene is present in all 23 species belonging to clade S1. Furthermore, it is also present in 95.2% of species in clade P2, except for *L. fluminis*, and in 13 out of the 20 species in clade P1, representing 65%. Finally, three species in S2 also carry the VanT gene: *L. ilyithenensis*, *L. kobayashii*, and *L. ryugenii* (Figure 1).

The gene VanW in the VanB cluster is even less common and is only present in *L. adleri* in P1 and in *L. koniambonensis* and *L. johnsonii* in P2. However, it has more representatives in clade S1 with eight species: *L. biflexa*, *L. bouyouniensis*, *L. yanagawae*, *L. levettii*, *L. ellinghausenii*, *L. wolbachii*, *L. congkakensis*, and *L. kanakyensis*. The most common VanW gene is the one present in the VanG cluster with the highest incidence in clade S1, except for *L. biflexa*, *L. bouyouniensis*, *L. levettii*, and *L. ellinghausenii*. Only 4 out of 20 species in clade P1 carry the VanW gene in the VanG cluster: *L. yasudae*, *L. stimsonii*, *L. gomenensis*, and *L. sanjuarensis*. Even less representation is shown in clade P2, where only *L. neocaledonica* and *L. wolffii* carry the sequence (Figure 1).

The gene VanY is the least represented across the genus, with 20%, 24%, and 30% incidence in clades P1, P2, and S1, respectively. Additionally, the VanY gene in the VanG cluster is the least represented of these genes and is only present in three species belonging to clade P2, *L. wolffii*, *L. fletcheri*, and *L. fluminis*. Finally, the genes VanW and VanY are absent altogether in clade S2 (Figure 1).

**Figure 2:**
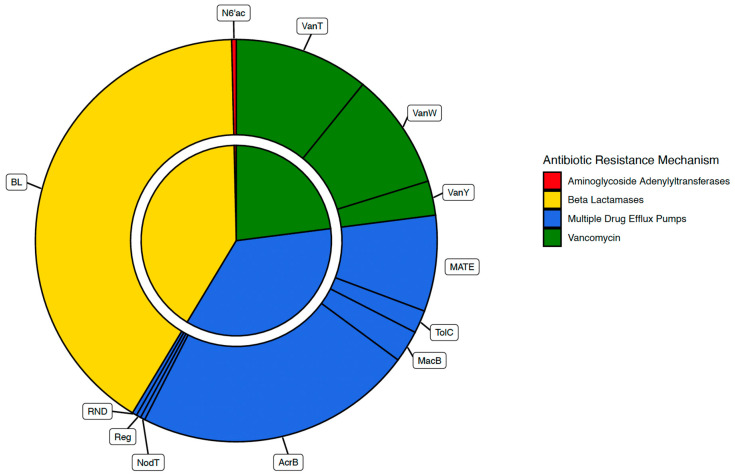
Diagram showing the proportion of each of the four mechanisms of antibiotic resistance found in *Leptospira* in the inner circle: beta-lactamases, vancomycin, efflux pumps, and aminotransferases. The outer circle shows the proportion of each of the 12 genes found that correspond to the four mechanisms: VanT, VanW, and VanY in vancomycin; MATE, TolC, MacB, AcrB, NodT, Reg, and RND in efflux pumps, beta-lactams; and N6′ac in aminotransferases.

Interestingly, the VanW gene in the VanG cluster is present up to threefold in *L. chreensis* and twofold in the other 14 species, all of them belonging to the same type of free-living saprophytes grouped into clade S1. This is the only case of repeated copies of a gene associated with antibiotic resistance to vancomycin found in *Leptospira* thus far. Clade S1 is composed of 23 species, from which 15 have repeated copies of the VanW gene in the VanG cluster, and the other four species possess single copies of the same gene. Furthermore, *L. yanagawae*, *L. wolbachii*, *L. congkakensis*, and *L. kanakyensis* carry VanW genes in both clusters. Although *L. biflexa*, *L. bouyouniensis*, *L. levettii*, and *L. ellinghausenii* miss the VanW gene in the VanG cluster, they do possess the VanW gene in the VanB cluster, making clade S1 the only group to have VanW genes in all species. (Figure 2).

The VanT gene is highly conserved, with the sequences averaging 1148 nucleotides. The shorter sequence is present in *L. fletcheri* with 1095 nucleotides, and the largest sequence is present in *L. broomii*, *L. inadai*, and *L. fainei* with 1254 nucleotides. Another highly conserved sequence is the VanY gene, with the largest range present in the VanB cluster, with 744 and 801 nucleotides in the smallest and largest sequences, respectively. On the other hand, the VanW gene shows more variations. Although the VanW gene in the VanB cluster tends to be conserved, *L. adleri* shows an unusual length in comparison to the other species with the same cassette. The VanW gene present in *L. adleri* comprises 1800 nucleotides, but the other 10 species have a range from 819 to 885 nucleotides. Regarding the VanW gene in the VanG cluster, it can be said that it is the most variable gene, with a minimum of 618 nucleotides in *L. congkakensis* and a maximum of 1875 nucleotides in *L. yasudae*, with an average of 881 nucleotides.

The VanT and VanY genes are equally conserved among the four clades of *Leptospira*, while the VanW gene shows high polymorphisms even among the clades. The largest sequences are present in clade P1, with *L. adleri*, *L. stimsonii*, and *L. sanjuarensis* having the shortest sequences with 1800 nucleotides each, and *L. yasudae* having the largest sequence with 1875 nucleotides. The clade P2, being the most conserved with four species that carry the gene, shows a constant number of 819 nucleotides. Finally, the highest variation in length is present in clade S1, with *L. congkakensis* having the shortest sequence with 618 nucleotides and *L. perdikensis* having the longest sequence with 894 nucleotides.

### 3.3. Resistance to Beta-Lactamases

Genes associated with resistance to beta-lactam antibiotics were found in all 69 species using RAST, with at least two copies of the gene in 25% of the species. In addition to that, 33% of species of *Leptospira* carry three copies of these genes, and 40% of the species carry four copies of genes associated with resistance to beta-lactam antibiotics, mostly present in clade S1. Additionally, *L. yanagawae* and *L. ellinghausenii* have five copies of these genes, the highest number of copies present among *Leptospira*. Finally, the lowest number of copies is present in P1, with 50%, 40%, and 20% having two, three, and four copies, respectively. Clade P2 shows a higher number of incidences of extra copies of these genes, with 29% of the species having two, 29% of the species having three copies, and 42% of the species carrying four copies of the gene. Moreover, 26% of the species in clade S1 have three copies, 48% have four copies, and only *L. terpstrae* carries two copies, in contrast to *L. yanagawae* and *L. ellinghausenii,* which have the highest number with five copies of the gene. Finally, in clade S2, three species have three copies, while two species have four copies.

These genes are also highly polymorphic, even among the four clades. The shortest and largest sequences in clade P1 are *L. adleri* and *L. stimsonii,* with 114 and 1380 nucleotides, respectively. The shortest sequence in clade P2 is carried by *L. broomii* with 108 nucleotides, which is also the shortest sequence among all species of the genus studied. On the other hand, the largest sequence is carried by *L. harrisiae* with 2007 nucleotides. Moreover, the shortest sequence present in S1 can be found in *L. ellinghausenii* with 261 nucleotides, while the largest is present in *L. abararensis* with 2049 nucleotides. Finally, *L. idonii* has four sequences of such genes, with two of them being the shortest and largest sequences, with 561 and 2100, respectively. At the same time, *L. idonii* is the species with the largest sequence of this type of gene across the entire genus of *Leptospira*.

### 3.4. Multidrug Resistance Efflux Pumps

Another mechanism of antibiotic resistance found using RAST was multidrug resistance efflux pumps, and seven different sequences were found to be associated with this specific mechanism as follows: multidrug and toxic compound extrusion proteins (MATE), efflux protein TolC, macrolide export ATP-binding proteins, acriflavine resistance proteins, NodT proteins, transcription regulators, and membrane fusion proteins. Interestingly, these proteins were absent in clade P1 but present in the other three clades.

Multidrug and toxic compound extrusion proteins were present in 17 species out of the 21 belonging to clade P2; 7 species have one copy of the gene, while 10 species have two copies of it. Meanwhile, *L. brenneri* is the only representative of clade S1 with two copies of the gene; ten more species only possess a single copy, and twelve species do not have the cassette. Finally, three species have a single copy of MATE-family proteins, and both *L. idonii* and *L. ryugenii* do not (Figure 1). The shortest sequences belong to *L. koniambonensis* and *L. perolatii*, both belonging to clade P2 with 1233 nucleotides, while the longest sequence is present in *L. brenneri* with 2079 nucleotides, which is also 41% larger than the second to last with 1473 present in *L. broomii.*

Efflux protein TolC was only found in single copies in *L. licerasiae*, *L. hartskeerlii*, *L. venezuelensis*, *L. selangorensis*, *L. haakeii*, *L. andrefontaineae*, *L. saintgironsiae*, *L. neocaledonica*, *L. johnsonii*, and *L. perolatii*, which belong to clade P2. No representatives of the other three clades were found to carry this specific sequence (Figure 1). The first nine species have sequences with 1476 nucleotides, and *L. perolatii* is the only species to have 1491 nucleotides.

*L. perolatii* is the only member of clade P2 that carries macrolide-export ATP-binding proteins. Furthermore, ten species belonging to clade S1, representing 39% of the group, also carry this cassette, with *L. mtsangambouensis* being the only species to have two copies across the entire genus. Finally, *L. ilyithenensis* and *L. kobayashii* are the two members of the five belonging to clade S2 found to carry these types of proteins (Figure 1). The shortest sequence is present in *L. mtsangambouensis,* with 705 nucleotides. Meanwhile, the rest of the species that carry this cassette have conserved sequences, with ten species having 1947 nucleotides and two species having 1953 nucleotides.

Sequences related to acriflavine resistance stand out in comparison to any other genes associated with efflux pumps, and they are the second most common across other mechanisms in the entire genus *Leptospira*. Although clade P1 has no incidence of acriflavine resistance genes, the other three clades show major incidences by showing the highest numbers of copies related to antibiotic resistance across the entire genus for all mechanisms discussed here. On the one hand, five species belonging to P2, twelve species to S1, and two species to S2 do not possess the genes associated with resistance to acriflavine. On the other hand, the lowest number of copies of such a gene is three, with incidences in *L. dzoumogneensis*, *L. venezuelensis*, *L. selangorensis*, *L. haakeii*, *L. saintgironsiae*, *L. neocaledonica*, *L. johnsonii*, *L. sarikeiensis*, and *L. fainei* in clade P2; *L. vanthielii*, and *L. meyeri* in clade S1, representing 14.5% and 3% across the entire genus, respectively. Furthermore, no species in clade S2 has three copies of the gene. Additionally, *L. hartskeerlii*, *L. koniambonensis*, *L. broomii*, and *L. inadai* in clade P2; *L. brenneri*, *L. terpstrae*, and *L. bandrabouensis* in clade S1; and finally, *L. ilyithenensis* and *L. kobayashii* in clade S2 show four copies of the gene related to acriflavine resistance, which corresponds to 6%, 4%, and 3%, respectively. While nine species across the entire genus possess four copies of this gene, seven species have five copies: *L. andrefontaineae* and *L. langatensis,* belonging to P2, *L. abararensis*, *L. noumeaensis*, *L. mtsangambouensis*, and *L. harrisiae,* belonging to S1, and *L. ognonensis,* from clade S2. Finally, the maximum number of copies of genes related to acriflavine resistance was found to be six, present only in *L. licerasiae* in clade P2 and in *L. bourretii* and *L. montravelensis* in clade S1 (Figure 1). This sequence is more polymorphic than the rest and is also related to efflux pumps. The shortest sequence is one of the six copies present in *L. licerasiae* belonging to clade P2, with 2667 nucleotides. The largest sequence is also present in clade P2 in *L. langatensis* with 4338 nucleotides. The average nucleotide count in these sequences is 3125 and there are no patterns among the three clades showing this cassette, so it can be said that the length is randomly distributed in all species having this sequence.

The other three proteins that were found to be related to efflux pumps are NodT proteins, transcription regulators, and membrane fusion proteins. Each of the previous proteins is found in only two species across the genus. *L. brenneri* and *L. noumeaensis*, with 1470 nucleotides each, both belonging to clade S1, have NodT proteins involved in secretion. Transcription regulators are present in clade P2 in *L. johnsonii,* with 582 nucleotides, and in *L. inadai,* with 723 nucleotides. Finally, membrane fusion proteins were found in *L. licerasiae* and *L. neocaledonica,* with 1437 and 1419 nucleotides, respectively, also belonging to clade P2.

### 3.5. Resistance to Aminoglycoside Adenylyltransferases

The least represented mechanism related to antibiotic resistance found in *Leptospira* is aminoglycoside adenylyltransferases, with only a 3% incidence across the entire genus. The gene N6′ac (aminoglycoside N6′-acetyltransferase) was found to be present in the most novel species, *L. sanjuarensis*, belonging to clade P1, with 357 nucleotides, and in *L. kanakyensis* from clade S1 with 528 nucleotides. This gene was not found in any species belonging to clades P2 and S2.

## 4. Discussion

*Mechanisms of Resistance*

In this research, 12 genes related to four antibiotic resistance mechanisms in *Leptospira* were identified and analyzed: beta-lactamases, vancomycin, efflux pumps, and aminotransferases. This study demonstrates the presence of genes related to antibiotic resistance in different species of the *Leptospira* genus.

As mentioned earlier, different studies have found susceptibility and resistance to some antibiotics in *Leptospira* in vitro. However, the results obtained from this research are novel and suggest a new approach to the study of antibiotic resistance in *Leptospira*. Susceptibility to vancomycin was first seen in *Leptospira* in 1980, potentially without the proper knowledge of the target Gram-stain for vancomycin. In fact, it is now known that vancomycin does not act properly on *Leptospira*; therefore, it raises the question of whether the lack of action was due to the presence of genes associated with antibiotic resistance to vancomycin or merely because the wrong antibiotic was used [33]. In 1988, another study found a streptomycin-resistant mutant of *L. biflexa* to be cross-resistant to dihydrostreptomycin but not to other antibiotics used in the study [34]. According to the current investigation, there is no evidence that any species of *Leptospira* is inherently resistant to streptomycin or dihydrostreptomycin. In Japan, 46 *Leptospira* isolates were studied, and the researchers found them to be sensitive to seven different antibiotics and resistant to the other seven, including vancomycin, which again raises the question of whether this is the result of the genes related to resistance to vancomycin found in the current study or only due to the use of the wrong antibiotic on *Leptospira* [35]. In 2018, five different antibiotics were tested on different strains of *L. santarosai, L. noguchii, and L. interrogans* isolated from cattle in Brazil, showing reduced susceptibility to tetracycline, which also does not match the genes found currently [36]. Finally, the most recent studies on susceptibly and resistance in *Leptospira* were published in 2020, where environmental, livestock, and human isolates were analyzed. These studies showed evidence to suggest resistance to trimethoprim and sulphamethoxazole and reduced susceptibility to penicillin, ceftriaxone, doxycycline, tetracycline, and six other antibiotics, with no direct relationship to any of the current mechanisms associated with antibiotic resistance found in any of the 69 *Leptospira* genomes [37,38].

The origin of the resistance mechanisms in *Leptospira* could be related to exposure to diverse environments and interactions with animal hosts. Antibiotic use practices in human and veterinary medicine could also have influenced the selection of resistant strains in this bacterium. It is important to consider how patterns of antibiotic use, both in healthcare and in animal husbandry, may have contributed to the development and spread of such genes in *Leptospira*. The clinical and public health impacts of these resistance mechanisms should not be underestimated. Antibiotic resistance in *Leptospira* could significantly complicate the treatment of leptospirosis, limiting the available therapeutic options and increasing the disease burden in humans. The implementation of effective control and prevention measures, as well as the promotion of practices for the responsible use of antibiotics, are essential to mitigate the risk of spreading resistant strains and to preserve the efficacy of current treatments.

Furthermore, the importance of analyzing antibiotic resistance in clades S1 and S2 should not be overlooked. Although these two clades are not pathogenic and do not pose a threat to other species, they could potentially exchange genes related to antibiotic resistance, especially considering the number of genes found in both clades and the number of copies present in them. *L. bourretii* and *L. montravelensis* are two of the three species with six copies of the gene AcrB. Although none of the mechanisms found in this study match the current course of treatment, it is necessary to study the origin of such genes to a greater extent to be able to establish prevention programs to reduce the further acquisition of genes capable of providing resistance to those antibiotics used nowadays to treat infection of leptospirosis. Interestingly, the free-living clades seem to have a higher incidence of genes associated with antibiotic resistance, probably due to the exposure to antibiotics used in farming together with runoff. However, the reasons for these acquisitions should be investigated through the analysis of genomes of more variants of the same species, depending on the geographic region.

In summary, this research has shed light on the presence of genes related to antibiotic resistance in the *Leptospira* genus. A detailed understanding of these mechanisms provides a solid foundation for the design of treatment and control strategies that address the challenges posed by resistance in this bacterium. Given the dynamic nature of antibiotic resistance, it is essential to continue to investigate and closely monitor the evolution and spread of these mechanisms in *Leptospira* and other relevant pathogens. Following this line of research, it is crucial to consider how the antibiotic resistance mechanisms identified in *Leptospira* may be influenced by exposure to antibiotics in moist soils and water sources. Resistance genes can be transferred horizontally between bacteria, suggesting the possibility that *Leptospira* may acquire these resistance mechanisms from other bacteria present in these environments. The coexistence and interaction with other resistant bacteria could be contributing factors in the acquisition of these resistance genes and their subsequent evolution in *Leptospira*.

Moreover, it is important to mention that the presence of these genes does not necessarily mean that the organism possesses the resistant phenotype. In fact, a whole series of genes is needed to confer functional resistance to a given antibiotic. To further study resistance in *Leptospira*, experiments on gene expression are needed. Although the previous studies hereby mentioned have used in vitro approaches, these studies have not taken into consideration the acquisition of the genes associated with antibiotic resistance. Therefore, the use of bioinformatics tools should precede further in vitro experimentation. Finally, it is also critical to take into consideration that this study has been performed by using only one strain of every species, which also has its limitations. In future studies, the research can be expanded to include all species and their related serovars with sequenced genomes to understand the dynamics of gene acquisition related to antibiotic resistance mechanisms. It will also be necessary to conduct gene expression studies and proteomic analyses to confirm the functionality of these mechanisms and rule out the possibility that these genes are remnants or non-functional.

## 5. Conclusions

In conclusion, this bioinformatics study reveals the presence of genes related to antibiotic resistance mechanisms in the *Leptospira* genus, highlighting the vital importance of expanding this analysis to all sequenced genomes of the species and serovars of the genus. Additionally, transcriptomic and proteomic studies should be conducted in parallel to provide experimental evidence of the functionality of these genes. Finally, this methodology could be applied for epidemiological surveillance of antibiotic resistance mechanisms being incorporated into the genomes of the different species and serovars that make up the *Leptospira* genus.

## Figures and Tables

**Table 1:** Described *Leptospira* species shown within subclades with their corresponding accession number, genome size (Mb), CG content (%), number of genes, proteins, and non-coding sequences annotated in the NCBI’s GeneBank.

Species Names	Clade	Accession Number	Genome Size (Mb)	GC (%)	Genes	Proteins	Non-Coding
*L. adleri*	P1	GCF_004770415.1	4.0	43.5	3507	3435	32
*L. ainazelensis*	P1	GCF_016918785.1	4.9	42.5	4448	4259	45
*L. ainlahdjerensis*	P1	GCF_016919175.1	4.8	42.5	4437	4236	42
*L. alexanderi*	P1	GCF_002009845.1	4.1	40.0	3756	3460	42
*L. alstonii*	P1	GCF_001569395.1	4.2	42.5	3854	3653	43
*L. barantonii*	P1	GCF_004770795.1	4.4	43.5	4165	4031	43
*L. borgpetersenii*	P1	GCF_003516145.1	4.0	40.0	3454	3223	44
*L. ellisii*	P1	GCF_002812215.1	4.1	47.5	4664	3727	41
*L. gomenensis*	P1	GCF_004770155.1	4.3	46.0	3931	3794	43
*L. interrogans*	P1	GCF_002073495.2	4.6	35.0	3776	3652	44
*L. kirschneri*	P1	GCF_000243695.2	4.4	35.5	3637	3399	43
*L. kmetyi*	P1	GCF_003722295.1	4.4	44.5	4021	3913	45
*L. mayottensis*	P1	GCF_004770855.1	4.0	39.5	3694	3385	44
*L. noguchii*	P1	GCF_000306255.2	4.7	35.5	4034	3798	44
*L. sanjuarensis*	P1	GCF_022267325.1	4.5	45.0	4157	3984	45
*L. santarosai*	P1	GCF_000313175.2	4.0	41.5	3611	3468	44
*L. stimsonii*	P1	GCF_004769715.1	5.0	42.5	4670	4498	43
*L. tipperaryensis*	P1	GCF_001729245.1	4.6	42.0	4153	4065	46
*L. weilii*	P1	GCF_006874765.1	4.4	40.5	3975	3738	44
*L. yasudae*	P1	GCF_003545865.1	4.5	45.5	4083	3990	45
*L. andrefontaineae*	P2	GCF_004770105.1	4.3	39.5	3958	3901	43
*L. broomii*	P2	GCF_000243715.2	4.4	42.5	3954	3881	47
*L. dzoumogneensis*	P2	GCF_004770895.1	4.1	40.5	3809	3750	40
*L. fainei*	P2	GCF_000306235.2	4.3	43.5	3866	3801	46
*L. fletcheri*	P2	GCF_004769195.1	3.7	47.0	3430	3372	44
*L. fluminis*	P2	GCF_004771275.1	3.7	47.5	3438	3378	44
*L. haakeii*	P2	GCF_002812045.1	4.2	39.5	3866	3797	42
*L. hartskeerlii*	P2	GCF_002811475.1	4.1	40.0	3749	3692	43
*L. inadai*	P2	GCF_001704175.2	4.6	44.5	4085	3980	49
*L. johnsonii*	P2	GCF_003112675.1	4.1	41.0	3781	3729	45
*L. koniambonensis*	P2	GCF_004769555.1	4.3	38.5	3992	3941	41
*L. langatensis*	P2	GCF_004770615.1	4.1	44.5	3755	3701	47
*L. licerasiae*	P2	GCF_000526875.1	4.2	41.0	3895	3827	49
*L. neocaledonica*	P2	GCF_002812205.1	4.2	40.0	3910	3861	43
*L. perolatii*	P2	GCF_002811875.1	4.0	42.0	3629	3554	46
*L. saintgironsiae*	P2	GCF_002811765.1	4.1	39.0	3792	3737	41
*L. sarikeiensis*	P2	GCF_004769615.1	4.4	44.5	3755	3701	47
*L. selangorensis*	P2	GCF_004769745.1	4.2	39.5	3881	3827	41
*L. semungkisensis*	P2	GCF_004770055.1	3.9	42.5	3627	3567	43
*L. venezuelensis*	P2	GCF_002150055.1	4.3	39.0	4020	3967	42
*L. wolffii*	P2	GCF_004770635.1	4.2	45.5	3854	3788	44
*L. abararensis*	S1	GCF_016918735.1	4.2	39.0	3979	3831	40
*L. bandrabouensis*	S1	GCF_004770555.1	4.2	37.5	3924	3876	42
*L. biflexa*	S1	GCF_000017685.1	4.0	38.5	3716	3658	43
*L. bourretii*	S1	GCF_004769655.1	4.3	38.0	3993	3938	43
*L. bouyouniensis*	S1	GCF_004770625.1	4.1	37.0	3833	3782	43
*L. brenneri*	S1	GCF_002812125.1	4.1	38.0	3863	3803	41
*L. chreensis*	S1	GCF_016919165.1	4.5	39.5	4200	4040	42
*L. congkakensis*	S1	GCF_004770225.1	4.0	38.0	3729	3682	41
*L. ellinghausenii*	S1	GCF_003114815.1	4.2	37	3950	3886	43
*L. harrisiae*	S1	GCF_002812135.1	4.1	37.5	3853	3797	43
*L. jelokensis*	S1	GCF_004769635.1	4.0	39.0	3703	3639	42
*L. kanakyensis*	S1	GCF_004769445.1	4.2	38.0	3975	3923	41
*L. kemamanensis*	S1	GCF_004769665.1	3.8	38.5	3545	3477	42
*L. levettii*	S1	GCF_002812185.1	4.3	37.0	4180	4011	43
*L. meyeri*	S1	GCF_004368965.1	4.2	38.0	3959	3905	42
*L. montravelensis*	S1	GCF_004769455.1	4.1	37.0	3839	3788	42
*L. mtsangambouensis*	S1	GCF_022267475.1	4.2	38.0	3946	3805	44
*L. noumeaensis*	S1	GCF_004770765.1	4.1	38.0	3849	3798	41
*L. perdikensis*	S1	GCF_004769575.1	4.0	38.5	3734	3684	41
*L. terpstrae*	S1	GCF_000332495.1	4.1	38.0	3869	3812	46
*L. vanthielii*	S1	GCF_004770365.1	4.1	39.0	3830	3773	41
*L. wolbachii*	S1	GCF_000332515.2	4.1	39.0	3856	3788	45
*L. yanagawae*	S1	GCF_004769275.1	4.0	38.0	3695	3641	41
*L. idonii*	S2	GCF_004770995.1	4.1	41.0	3828	3767	41
*L. ilyithenensis*	S2	GCF_004771005.1	4.2	40.5	3976	3887	42
*L. kobayashii*	S2	GCF_003114835.2	4.3	40.5	3948	3888	43
*L. ognonensis*	S2	GCF_004770745.1	4.0	39.5	3746	3674	41
*L. ryugenii*	S2	GCF_003114855.1	4.0	39.5	3720	3659	42

## Data Availability

The data presented in this study are available at (https://www.ncbi.nlm.nih.gov/datasets/genome/?taxon=171) and (https://ucesedu-my.sharepoint.com/:u:/g/personal/pineda_gomez_uces_edu_co/EdIYCEvF-JJFkWFZZ2fk2eIBc-qptft2ZKQn5IA99WWbjA).
